# Gait Analysis in Progressive Supranuclear Palsy Phenotypes

**DOI:** 10.3389/fneur.2021.674495

**Published:** 2021-06-10

**Authors:** Marina Picillo, Carlo Ricciardi, Maria Francesca Tepedino, Filomena Abate, Sofia Cuoco, Immacolata Carotenuto, Roberto Erro, Gianluca Ricciardelli, Michela Russo, Mario Cesarelli, Paolo Barone, Marianna Amboni

**Affiliations:** ^1^Department of Medicine, Surgery and Dentistry, Center for Neurodegenerative Diseases (CEMAND), University of Salerno, Fisciano, Italy; ^2^Department of Electrical Engineering and Information Technology, University of Naples “Federico II”, Naples, Italy; ^3^Istituti Clinici Scientifici Maugeri Istituto di Ricovero e Cura a Carattere Scientifico (IRCCS), Telese Terme, Italy; ^4^Department of Medicine, Azienda Ospedaliera Universitaria OO. RR. San Giovanni di Dio e Ruggi D'Aragona, Salerno, Italy; ^5^Istituto di Diagnosi e Cura (IDC) Hermitage-Capodimonte, Naples, Italy

**Keywords:** progressive supranuclear palsy, gait, phenotype, subtype, gait analysis

## Abstract

The objective of the present study was to describe gait parameters of progressive supranuclear palsy (PSP) phenotypes at early stage verifying the ability of gait analysis in discriminating between disease phenotypes and between the other variant syndromes of PSP (vPSP) and Parkinson's disease (PD). Nineteen PSP (10 PSP-Richardson's syndrome, five PSP-parkinsonism, and four PSP-progressive gait freezing) and nine PD patients performed gait analysis in single and dual tasks. Although phenotypes showed similar demographic and clinical variables, Richardson's syndrome presented worse cognitive functions. Gait analysis demonstrated worse parameters in Richardson's syndrome compared with the vPSP. The overall diagnostic accuracy of the statistical model during dual task was almost 90%. The correlation analysis showed a significant relationship between gait parameters and visuo-spatial, praxic, and attention abilities in PSP-Richardson's syndrome only. vPSP presented worse gait parameters than PD. Richardson's syndrome presents greater gait dynamic instability since the earliest stages than other phenotypes. Computerized gait analysis can differentiate between PSP phenotypes and between vPSP and PD.

## Introduction

Progressive supranuclear palsy (PSP) is a rare, rapidly progressive, neurodegenerative disease characterized by dysfunction in four core domains including ocular motor function, postural instability, akinesia, and cognition (1). The diverse combination of core clinical features is a determinant for the attribution of the clinical phenotype of disease (1). While PSP-Richardson's syndrome (PSP-RS) is the most common clinical phenotype, other distinct variants of the disease (vPSP) have been described, each featured by a specific predominant symptom (1). Although specific algorithms have been developed (1, 2), evidence shows that available clinical and radiological assessments do not support the differentiation of the PSP phenotypes (3–5).

Gait analysis is a tridimensional, computerized, and non-invasive investigation of walking and has been used in Parkinson's disease (PD) with different objectives including investigation of pathophysiological mechanisms underpinning the disease, evaluation of treatment outcomes, and algorithm implementation for PD diagnosis and staging (6, 7). Irrespective of the phenotype, PSP patients feature a profound gait dysfunction compared with other movement disorders (8–11). To date, there is no study comparing quantitative gait parameters between PSP phenotypes.

The aims of the present study were (1) to describe quantitative gait parameters of PSP phenotypes at early stages and (2) to verify the ability of gait analysis, performed with a motion capture system, in discriminating between PSP-RS and vPSP. Additional objectives were to describe the cognitive profile of PSP phenotypes and its correlation with gait parameters as well as to verify the ability of gait parameters in differentiating between vPSP and a population of PD patients matched for age, sex distribution, and disease duration.

## Methods

### Patients and Clinical Evaluation

Nineteen patients with probable PSP according to the Movement Disorder Society (MDS) criteria were included in the present analysis (1). Detailed information on enrollment and application of the PSP diagnostic criteria is available elsewhere (3–5). Ten qualified for PSP-RS (52.6%), while the remaining presented vPSP (five PSP with predominant parkinsonism and four PSP with predominant gait freezing) (Supplementary File 1 shows fulfillment of core features for each patient).

Specific exclusion criteria for the present study were as follows: gait requiring assistance (with cane, walker, or a person); dementia according to the *Diagnostic and Statistical Manual of Mental Disorders* (Fifth Edition) (DSM-V) criteria; and clinically significant comorbidities, including other neurologic disorders, orthopedic diseases, or cardiovascular/respiratory diseases.

Motor features were evaluated with the PSP rating scale (PSP-rs) (12). Also, PSP-rs gait/midline subscore was considered (12).

Cognitive abilities were screened with the Montreal Cognitive Assessment (MOCA). Memory domain was investigated with the immediate and delayed recall scores of the Rey auditory verbal learning test (15-RAWLT) and the prose memory test. Attention domain was explored through the trail-making test (TMT) and the short version of the Stroop interference test. Executive functions were assessed with phonological and semantic verbal fluency tests. Visuo-spatial functions were tested with the constructional apraxia test and Benton orientation line test (BJLO). Praxic abilities were explored through the bucco-facial and ideomotor apraxia tests (4, 13).

In addition, in order to compare gait features between vPSP and PD, a population of nine PD patients (75% men; age = 65 ± 3.42 years; disease duration = 2 ± 0.71 years) was recruited.

The study was approved by the local ethics committee, and all patients were included upon signing the written informed consent.

### Gait Analysis

Gait analysis was performed for all subjects through the motion capture system of BTS Bioengineering (Quincy, MA, USA). The SMART DX is an optical system equipped with six infrared cameras, two video cameras, two force plates, a set of passive markers, and an elaborator. The Davis protocol was used for all subjects, including the following phases: (1) taking anthropometric measures (i.e., height, weight, and length of the leg); (2) positioning of 22 reflective markers on specific points on the body; (3) standing phase, which requires the patient to standing on the force plate; and (4) walking phase on a path of 10 m.

All patients were evaluated on the straight pathway during different tasks, each one performed four times: (1) GAIT: normal gait, namely, the single task; and (2) COG: walking while serial subtracting 7 s starting from 100, namely, the dual task.

Before the trials, all participants were trained to walk at a normal pace, at their usual speed, without any suggestion on prioritizing walking or calculating. Further details on the procedure are described elsewhere (14, 15).

### Statistical Analysis

A comparison between groups was performed with univariate non-parametric analysis (i.e., the Mann–Whitney test), while Spearman's correlation analysis was used to assess the association among the variables.

Binary logistic regression was used to produce models capable of classifying PSP phenotypes from spatial and temporal parameters of gait. Multicollinearity was checked by computing the coefficient of correlation, while the a-dimensional Cook's distance and the center leverage value were computed in order to identify the outliers; the distance among the points on a graph Cook's distance (x axis) and the center leverage value (y axis) was evaluated. With such a standard procedure, three outliers were overall identified (one for the GAIT task and two for the COG task). Indeed, Cook's distance is considered a summary measure of influence since a large value of Cook's distance indicates an influential observation (16). Similarly, observations that are far from the average covariate pattern are considered to have a high center leverage value. They are usually employed in literature for the statistical detection of outliers (17).

Then, the odds ratios with a confidence interval of 95% and the relative *p*-values were provided per each variable included in the models. Moreover, the Hosmer–Lemeshow goodness-of-fit test was computed to evaluate whether or not the observed event rates match expected event rates in the subgroups of the model population. Finally, the interference of the cognitive dual task was computed per each gait variable with the following equation (1):

$$\begin{array}{l} Interference=\;\frac{|DT-ST{|}^{*}100}{ST} \end{array}$$

where DT stands for dual task and ST for single task. Then, the Mann–Whitney test was used to verify the difference between the interferences of PSP-RS and vPSP groups. Significance level was set at *p* < 0.05. Statistical analysis was performed with IBM SPSS v.25.

## Results

PSP phenotypes did not disclose differences in demographic nor clinical variables (Table 1).

**Table 1:** Demographic, clinical and cognitive features according to PSP phenotype.

	**PSP-RS**	**vPSP**	***p***
**Demographics**			
Age, years	69.99 ± 7.66	66.56 ± 5.94	0.549
M/W, *N*	5/5	6/3	0.549
Education, years	10.1 ± 6.24	9.78 ± 5.4	0.906
**Clinical features**			
Disease duration, years	2.5 ± 1.17	2.33 ± 1	0.745
PSP-rating scale	34.9 ± 13.8	28.33 ± 9.38	0.247
PSP-rating scale gait/midline subscore	7.7 ± 4	6.77 ± 2.77	0.571
**Cognitive features**			
MOCA	15.33 ± 5.75	19.56 ± 4.28	0.161
**Memory domain**			
15-RAWLT-immediate	28.20 ± 13.06	29.11 ± 9.44	0.720
15-RAWLT-delayed	6.00 ± 3.77	7.64 ± 1.91	0.278
Prose memory	10.41 ± 4.81	9.48 ± 4.68	0.661
**Attention domain**			
TMT part A	148.75 ± 67.04	64.78 ± 39.22	**0.036**
TMT part B	298.43 ± 111.86	170.00 ± 64.40	**0.021**
TMTpart B–part A	168.43 ± 89.21	121.00 ± 55.84	0.336
Stroop time	29.28± 19.37	23.78 ± 19.46	0.863
Stroop errors	9.89 ± 10.98	5.67 ± 8.66	0.190
**Executive domain**			
Phonological fluency	12.33 ± 7.16	23.00 ± 6.10	**0.006**
Semantic fluency	21.00 ±10.68	29.33 ± 7.55	0.077
**Visuo-spatial domain**			
Constructional apraxia	5.40 ± 3.84	9.78 ± 3.31	**0.017**
BJLO	12.56 ± 6.64	13.78 ± 7.61	0.730
**Praxic abilities**			
Bucco-facial	15.89 ± 3.37	16.50 ± 2.83	0.673
Right ideomotor	55 ± 15.80	66.89 ± 5.58	**0.011**
Left ideomotor	49.33 ± 24.33	63.44 ± 4.88	0.222

*Values are given in mean ± standard deviation unless otherwise specified*.

*PSP phenotypes did not differ for years of education (p = 0.905)*.

*15-RAWLT, the Rey auditory verbal learning test; BJLO, Benton's Judjement of Line Orientation; M, men; MOCA, Montreal Cognitive Assessment battery; N, number; PSP-RS, Progressive Supranuclear Palsy with Richardson's syndrome; TMT, Trial Making Test; vPSP, the other variant syndromes of Progressive Supranuclear Palsy; W, women*.

*Significant results are highlighted in bold*.

Gait spatial and temporal parameters for both GAIT and COG tasks are reported in Table 2.

**Table 2:** Univariate statistical analysis comparing all gait parameters between PSP-RS and vPSP.

**Gait variables**	**Measure**	**GAIT task**			**COG dual task**		
		**PSP-RS**	**vPSP**	***p***	**PSP-RS**	**vPSP**	***p***
Cycle duration	s	1.39 ± 0.24	1.17 ± 0.17	**0.018**	1.54 ± 0.26	1.25 ± 0.23	**0.020**
Stance duration	s	0.88 ± 0.16	0.73 ± 0.12	**0.034**	1.03 ± 0.18	0.81 ± 0.20	**0.020**
Swing duration	s	0.51 ± 0.09	0.44 ± 0.06	0.095	0.51 ± 0.09	0.44 ± 0.05	0.135
Variability of swing's duration	s	0.13 ± 0.26	0.05 ± 0.03	0.382	0.07 ± 0.02	0.06 ± 0.03	0.678
Stance phase	%	63.39 ± 2.98	62.11 ± 2.47	0.277	66.78 ± 2.67	63.94 ± 4.09	**0.031**
Swing phase	%	36.53 ± 2.91	37.66 ± 2.89	0.345	33.23 ± 2.67	36.06 ± 4.09	**0.031**
Single support phase	%	36.60 ± 2.99	37.65 ± 2.94	0.345	33.23 ± 2.67	36.07 ± 4.09	**0.031**
Double support phase	%	14.79 ± 4.90	15.40 ± 8.65	0.602	17.26 ± 3.02	17.78 ± 7.34	0.571
Mean velocity	m/s	0.61 ± 0.14	0.78 ± 0.35	0.247	0.43 ± 0.10	0.65 ± 0.31	0.098
Mean velocity	% height/s	38.05 ± 8.15	46.53 ± 20.61	0.345	27.07 ± 6.29	38.87 ± 18.40	0.135
Cadence	Steps/min	89.51 ± 17.04	104.20 ± 14.40	**0.018**	80.66 ± 14.34	98.99 ± 16.70	**0.025**
Cycle length	m	0.82 ± 0.12	0.86 ± 0.30	0.917	0.65 ± 0.15	0.75 ± 0.29	0.521
Cycle length	% height	51.13 ± 7.04	52.48 ± 18.00	0.972	40.26 ± 9.07	46.09 ± 17.40	0.624
Step length	m	0.39 ± 0.09	0.41 ± 0.16	0.917	0.30 ± 0.10	0.38 ± 0.15	0.343
Variability of the length of step	m	0.27 ± 0.71	0.52 ± 1.34	0.651	0.33 ± 0.76	0.07 ± 0.03	0.069
Step width	m	0.11 ± 0.05	0.10 ± 0.04	0.508	0.13 ± 0.06	0.09 ± 0.05	0.157

*Data are shown in mean ± standard deviation*.

*PSP-RS, Progressive Supranuclear Palsy with Richardson's syndrome; vPSP, the other variant syndromes of Progressive Supranuclear Palsy*.

*Significant results are highlighted in bold*.

As regards GAIT task, PSP-RS showed worse gait parameters than did vPSP. In detail, PSP-RS exhibited reduced cadence and increased cycle duration (*p* = 0.018), mainly due to longer stance duration (*p* = 0.034).

As regards COG task, PSP-RS continued to roughly show the same gait features displayed during the single task. In addition, PSP-RS showed increased stance phase and reduced swing phase (*p* = 0.031), both of them expressed as percentage of the gait cycle. Also, there was a trend for significance for greater variability in step length (*p* = 0.069) and lower velocity (*p* = 0.098) in PSP-RS.

The results of the binary logistic regression aimed at distinguishing PSP phenotypes are shown in Table 3 for both tasks. The stance phase and the cadence were statistically significant in both models.

**Table 3:** Gait variables distinguishing PSP phenotypes according with the multinomial logistic regression.

	**GAIT**		**COG**	
**Gait variables**	**OR (95% CI)**	***p***	**OR (95% CI)**	***p***
Stance phase	1.143 (1.018–1.284)	**0.024**	1.227 (1.023–1.471)	**0.027**
Cadence	0.917 (0.850 – 0.989)	**0.026**	0.866 (0.758 – 0.989)	**0.033**

*CI, confidence intervals; OR, odds ratio; PSP, Progressive Supranuclear Palsy*.

*Significant results are highlighted in bold*.

As for the GAIT task, one outlier was removed; the overall accuracy of the model was 75%, while the capacity to detect PSP-RS and vPSP was 72.7 and 77.8%, respectively. The Hosmer–Lemeshow test had a *p*-value of 0.287.

As regards the COG task, two outliers were removed; the overall accuracy of the model was 88.9%, while the capacity to detect PSP-RS and vPSP was 90.9 and 85.7%, respectively. The Hosmer–Lemeshow test had a *p*-value of 0.164.

As for cognitive performances, PSP-RS compared with vPSP disclosed worse performances in the attention, executive, and visuo-spatial domains as well as worse praxic abilities (Table 1).

Table 4 shows that interference of the cognitive dual task was significant only for step length (greater per PSP-RS) and swing duration (greater for vPSP) (*p* < 0.05).

**Table 4:** Interference of the cognitive dual task by disease phenotype.

**Gait variables**	**PSP-RS**	**vPSP**	***P***
Cycle duration	12.80 ± 10.9	9.97 ± 7.03	0.862
Stance duration	19.81 ± 14.42	14.40 ± 10.03	0.464
Swing duration	6.58 ± 4.87	7.30 ± 4.68	0.651
Variability of swing's duration	30.20 ± 40.93	75.00 ± 68.99	**0.041**
Stance phase	5.96 ± 3.47	4.07 ± 3.86	0.193
Swing phase	9.81 ± 5.37	7.69 ± 7.52	0.277
Single support phase	9.98 ± 5.27	7.84 ± 7.55	0.310
Double support phase	33.99 ± 19.7	34.42 ± 35.60	0.602
Mean velocity (m/s)	27.81 ± 19.53	20.97 ± 14.32	0.464
Mean velocity (% height/s)	27.81 ± 16.26	23.97 ± 16.78	0.754
Cadence	10.66 ± 8.13	8.46 ± 5.39	0.651
Cycle length	21.56 ± 11.66	17.95 ± 13.82	0.345
Cycle length	21.56± 11.74	18.46 ± 13.79	0.464
Step length	22.76 ± 12.18	19.45 ± 14.89	0.464
Variability of the length of step	122.68 ± 148.8	37.60 ± 34.56	**0.023**
Step width	18.87 ± 11.73	15.31 ± 13.33	0.554

*Data are shown in mean ± standard deviation*.

*PSP-RS, Progressive Supranuclear Palsy with Richardson's syndrome; vPSP, the other variant syndromes of Progressive Supranuclear Palsy*.

*Significant results are highlighted in bold*.

The correlation analysis between COG parameters and cognitive tests for PSP phenotypes is shown in Table 5. As for PSP-RS, constructional apraxia and right ideomotor apraxia presented an inverse relationship with cycle and swing duration and a direct correlation with cadence (*p* < 0.05). TMT parts A and B showed a direct correlation with swing duration and cycle duration, respectively (*p* < 0.05). No significant correlations were shown for vPSP.

**Table 5:** Correlation analysis between COG parameters and cognitive tests for PSP-RS.

	**Constructional apraxia**	**TMT part A**	**TMT part B**	**Right ideomotor apraxia**
Cycle duration	−0.730 *P* = **0.017**	0.659 *P* = 0.076	0.714 *P* = **0.047**	−0.835 *P* = **0.003**
Swing duration	−0.681 *P* = **0.030**	0.826 *P* = **0.011**	0.619 *P* = 0.102	−0.701 *P* = **0.024**
Cadence	0.689 *P* = **0.027**	−0.620 *P* = 0.101	−0.671 *P* = 0.069	0.813 *P* = **0.004**

*Significant results are highlighted in bold*.

Differences in spatial and temporal parameters for both GAIT and COG tasks between vPSP and PD are reported in Table 6. As regards GAIT task, vPSP showed worse gait parameters than did PD. In detail, vPSP presented longer double support phase (*p* = 0.019), reduced velocity (*p* = 0.011) and cycle length (*p* = 0.014), and higher variability of the length of the step (*p* = 0.024). As regards COG task, vPSP continued to perform worse in the same gait features as in the GAIT task. In addition, vPSP showed longer swing duration (*p* = 0.006).

**Table 6:** Univariate statistical analysis comparing all gait parameters between PD and vPSP.

**Gait variables**	**Measure**	**GAIT task**			**COG dual task**		
		**PD**	**vPSP**	***p***	**PD**	**vPSP**	***p***
Cycle duration	s	1.08 ± 0.12	1.17 ± 0.17	0.190	1.18 ± 0.14	1.25 ± 0.23	0.161
Stance duration	s	0.65 ± 0.08	0.73 ± 0.12	0.094	0.69 ± 0.09	0.81 ± 0.20	0.136
Swing duration	s	0.43 ± 0.04	0.44 ± 0.06	0.666	0.29 ± 0.10	0.44 ± 0.05	**0.006**
Variability of swing's duration	s	0.03 ± 0.01	0.05 ± 0.03	0.063	0.09 ± 0.06	0.06 ± 0.03	0.190
Stance phase	%	60.18 ± 1.48	62.11 ± 2.47	0.113	61.27 ± 1.77	63.94 ± 4.09	0.050
Swing phase	%	39.89 ± 1.48	37.66 ± 2.89	0.113	38.42 ± 1.78	36.06 ± 4.09	0.094
Single support phase	%	39.90 ± 1.49	37.65 ± 2.94	0.113	38.41 ± 1.78	36.07 ± 4.09	0.094
Double support phase	%	10.02± 1.45	15.40 ± 8.65	**0.019**	11.50 ± 1.49	17.78 ± 7.34	**0.008**
Mean velocity	m/s	1.07 ± 0.10	0.78 ± 0.35	**0.011**	0.94 ± 0.18	0.65 ± 0.31	**0.050**
Mean velocity	% height/s	65.18 ± 7.56	46.53 ± 20.61	**0.011**	58.42 ± 10.84	38.87 ± 18.40	**0.040**
Cadence	Steps/min	112.05 ± 11.69	104.20 ± 14.40	0.190	109.11 ± 12.88	98.99 ± 16.70	0.190
Cycle length	m	1.14 ± 0.10	0.86 ± 0.30	**0.014**	1.05 ± 0.17	0.75 ± 0.29	**0.050**
Cycle length	%height	69.92 ± 4.97	52.48 ± 18.00	**0.019**	64.09 ± 8.71	46.09 ± 17.40	0.077
Step length	m	0.54 ± 0.11	0.41 ± 0.16	0.063	0.41 ± 0.17	0.38 ± 0.15	1.00
Variability of the length of step	m	0.21 ± 0.56	0.52 ± 1.34	**0.024**	0.21 ± 0.27	0.07 ± 0.03	0.077
Step width	m	0.08 ± 0.02	0.10 ± 0.04	0.190	0.07 ± 0.02	0.09 ± 0.05	0.136

*Data are shown in mean ± standard deviation*.

*vPSP, the other variant syndromes of Progressive Supranuclear Palsy from Richardson's syndrome; PD, Parkinson's disease*.

*Significant results are highlighted in bold*.

## Discussion

The present study shows quantitative gait parameters in PSP phenotypes at early stages. Herein, we demonstrate that PSP-RS patients show increased measures of dynamic instability compared with vPSP, mainly during a dual task (COG task).

PSP phenotypes were recently defined by the MDS as the combination of the differential involvement of disease core domains (1). Albeit few PSP phenotypes can be different at a clinical description level, multiple MDS core features can be applied to the same patient; and in the majority of cases, only the application of the artificial MDS algorithm allows the phenotypic subtyping (Supplementary File 1) (1, 2). In line with this scenario, evidence shows that available clinician-based assessments fail to detect major differences between phenotypes (3, 4). Similarly, objective midbrain-based radiologic measures can effectively differentiate PSP from PD or healthy controls but, again, fail to recognize PSP-RS from vPSP (5). As such, no objective biomarker is available to differentiate *in vivo* between PSP phenotypes.

In line with previous data (3–5), both PSP-RS total score and gait/midline subscore were similar in PSP-RS and vPSP in our study. Nevertheless, the objective measure of gait parameters presented significant differences among PSP phenotypes and disclosed worse gait performances in PSP-RS. Such difference was subclinical, as it was not detected with the PSP-RS total score or the gait/midline subscore. Moreover, the binary logistic regression applied on gait data showed acceptable diagnostic accuracy profile and a remarkable capacity to detect the phenotypes, particularly when using gait features during COG task. The highest diagnostic accuracy was shown during COG task, suggesting that dual task may represent a valid tool to distinguish between PSP phenotypes. However, when considering the equation, there is a differential interference of cognitive dual task on gait parameters according to the disease phenotype.

As for the pathophysiological significance of our findings, we can speculate that the greater subclinical gait dysfunction disclosed by PSP-RS may be underpinned by a greater involvement of midbrain and frontal circuits involved in gait control since the earliest stages of the disease. As such, a cognitive dual task is considered a proxy measure of frontal cognitive reserve. Thus, a suboptimal performance during a cognitive dual task suggests a reduced frontal reserve that, if compensated during a single task, collapses under such a challenging sensitizing tool (7). Also, our data suggest that the most affected parameter by the interference of the cognitive dual task in PSP-RS is the step length. Such interpretation is supported by the worse attention and executive functions displayed by PSP-RS compared with vPSP in spite of comparable MOCA scores. Confirming previous data on PD (6, 18), our findings support the evidence that cognitive loading exerts a detrimental effect on gait performance in PSP patients, the magnitude of which may be in part related to the underlying cognitive dysfunction. Furthermore, PSP-RS showed worse visuo-spatial functions in line with a growing body of evidence supporting the importance of visual information processing during the generation of motor plans and in the control of gait in PD (6, 19, 20). On the other hand, in vPSP, the most affected parameter by the interference of the cognitive dual task was swing duration, suggesting a possible different relationship between cognitive abilities and gait performance in the other phenotypes of the disease. However, as vPSP includes a heterogeneous group of patients (PSP with predominant parkinsonism and PSP with predominant gait freezing), more data are needed before drawing conclusions on this specific issue.

The correlation analysis further supported the notion of a greater influence of cognitive profile on COG task in PSP-RS compared with vPSP. In detail, specific gait parameters presented a significant correlation with visuo-spatial, praxic, and attention performances, while no such a relationship was shown for vPSP.

Moreover, as expected, several gait parameters in both GAIT and COG tasks were able to differentiate between vPSP and an age-, sex-, and disease duration-matched population of PD patients, further supporting the role of tridimensional, computerized, and non-invasive investigation of walking as a tool improving diagnostic accuracy. This is in line with previous data using inertial measurement units (IMUs), which employ portable accelerometers, gyroscopes, and magnetometers (21–23). Although IMUs provide reliable measurement of gait in a practical way, a motion capture system, as used in our study, is considered the gold standard for gait analysis due to its operating principle (it employs infrared cameras and passive markers to perform a tridimensional reconstruction of patients while walking). Moreover, the protocol used with IMUs is different from the Davis protocol adopted in the present study. Such different protocol explains the lack of numerous variables in our study compared with IMU-based studies. Therefore, we can only conclude that our data are in line with previous studies as regards spatial and temporal parameters of gait (19–21).

Ours is a preliminary, proof-of-concept study aimed at verifying if quantitative gait parameters can distinguish between PSP phenotypes. Future studies should further evaluate cognitive and imaging correlates of gait dysfunction in PSP phenotypes.

The present study has limitations. First, we recognize that the sample size is relatively small, which can particularly affect the results of the binary logistic regression. Furthermore, we only focused on gait parameters and emphasized dynamic instability while balance parameters were neglected. Also, in line with the request to walk on a straight pathway, no freezing episode occurred, as our gait protocol was not designed to elicit such phenomenon. Then, in spite of the accurate clinical characterization of our patients, we recognize that the lack of neuroimaging correlates does not allow us to draw firm conclusions on the pathophysiology of our findings. Notwithstanding, our study suggests that quantitative gait assessment may represent a potential biomarker for distinguishing PSP phenotypes and vPSP from PD. Furthermore, the present pilot study may pave the way for future developments with machine learning approaches on larger samples of PSP patients.

In conclusion, herein, we show that PSP-RS presents greater gait dynamic instability since the earliest stages of disease compared with vPSP. In addition, our findings indicate that gait quantitative evaluation can help to distinguish PSP-RS from vPSP.

## Data Availability Statement

The raw data supporting the conclusions of this article will be made available by the corresponding author, upon reasonable request.

## Ethics Statement

The studies involving human participants were reviewed and approved by Campania sud. The patients/participants provided their written informed consent to participate in this study.

## Author Contributions

Statistical analysis was conducted by CR. All authors contributed to the article and approved the submitted version.

## Conflict of Interest

MP has been supported by the Michael J. Fox Foundation for Parkinson's research. PB received consultancies as a member of the advisory board for Zambon, Lundbeck, UCB, Chiesi, Abbvie, and Acorda. RE received consultancies from Zambon and honoraria from TEVA. The remaining authors declare that the research was conducted in the absence of any commercial or financial relationships that could be construed as a potential conflict of interest.

## References

[1] Hoglinger GH Respondek G Stamelou M Kurz C Josephs KA Lang AE. Clinical diagnosis of progressive supranuclear palsy: the movement disorder society criteria. Mov Disord. (2017) 32:853–64. 10.1002/mds.26987 28467028 PMC5516529

[2] Grimm MJ Respondek G Stamelou M Arzberger T Ferguson L Gelpi E. How to apply the movement disorder society criteria for diagnosis of progressive supranuclear palsy. Mov Disord. (2019) 34:1228–32. 10.1002/mds.27666 30884545 PMC6699888

[3] Picillo M Erro R Cuoco S Tepedino MF Manara R Pellecchia MT. MDS PSP criteria in real-life clinical setting: motor and cognitive characterization of subtypes. Mov Disord. (2018) 33:1361–5. 10.1002/mds.27408 29984518

[4] Picillo M Cuoco S Tepedino MF Cappiello A Volpe G Erro R. Motor, cognitive and behavioral differences in MDS PSP phenotypes. J Neurol. (2019) 266:1727–35. 10.1007/s00415-019-09324-x 30989369

[5] Picillo M Tepedino MF Abate F Erro R Ponticorvo S Tartaglione S. Midbrain MRI assessments in progressive supranuclear palsy subtypes. J Neurol Neurosurg Psychiatry. (2020) 91:98–103. 10.1136/jnnp-2019-321354 31527182

[6] Amboni M Barone P Iuppariello L Lista I Tranfaglia R Fasano A. Gait patterns in Parkinsonian patients with or without mild cognitive impairment. Mov Disord. (2012) 27:1536–43. 10.1002/mds.25165 23032876

[7] Amboni M Iuppariello L Iavarone A Fasano A Palladino R Rucco R. Step length predicts executive dysfunction in Parkinson's disease: a 3-year prospective study. J Neurol. (2018) 265:2211–20. 10.1007/s00415-018-8973-x 30014240

[8] Amano S Skinner JW Lee HK Stegemoller EL Hack N Akbar U. Discriminating features of gait performance in progressive supranuclear palsy. Parkinson Relat Disord. (2015) 21:888–93. 10.1016/j.parkreldis.2015.05.017 26032992

[9] Ricciardi C Amboni M De Santis C Improta G Volpe G Iuppariello L. Using gait analysis' parameter to classify parkinsonism: a data mining approach. Comput Methods Programs Biomed. (2019) 180:105033. 10.1016/j.cmpb.2019.105033 31445485

[10] Gaßner H Raccagni C Eskofier BM Klucken J Wenning GK. The diagnostic scope of sensor-based gait analysis in atypical parkinsonism: further observations. Front Neurol. (2019) 10:5. 10.3389/fneur.2019.00005 30723450 PMC6349719

[11] Raccagni C Gassner H Eschlboeck S Boesch S Krismer F Seppi K. Sensor-based gait analysis in atypical parkinsonian disorders. Brain Behav. (2019) 8:e00977. 10.1002/brb3.977 29733529 PMC5991583

[12] Golbe LI Ohman-Strickland PA. A clinical rating scale for progressive supranuclear palsy. Brain. (2007) 130:1552–65. 10.1093/brain/awm032 17405767

[13] Santangelo G Cuoco S Pellecchia MT Erro R Barone P Picillo M. Comparative cognitive and neuropsychiatric profiles between Parkinson's disease, multiple system atrophy and progressive supranuclear palsy. J Neurol. (2018) 265:2602–13. 10.1007/s00415-018-9038-x 30178175

[14] Ricciardi C Amboni M De Santis C Ricciardelli G Improta G Cesarelli G. Classifying patients affected by Parkinson's disease into freezers or non-freezers through machine learning. In: 2020 IEEE International Symposium on Medical Measurements and Applications (MeMeA). (2020). 10.1109/MeMeA49120.2020.9137317

[15] Ricciardi C Amboni M De Santis C Ricciardelli G Improta G D'Addio G. Machine learning can detect the presence of mild cognitive impairment in patients affected by Parkinson's disease. In: 2020 IEEE International Symposium on Medical Measurements and Applications (MeMeA). (2020). 10.1109/MeMeA49120.2020.9137301

[16] Cook RD. Detection of influential observation in linear regression. Technometrics. (1977) 19:15–8. 10.1080/00401706.1977.10489493

[17] Zhang Z. Residuals and regression diagnostics: focusing on logistic regression. Ann Translat Med. (2016) 4:195. 10.21037/atm.2016.03.36 27294091 PMC4885900

[18] Yogev G Giladi N Peretz C Springer S Simon ES Hausdorff JM. Dual tasking, gait rhythmicity and Parkinson's disease: which aspects of gait are attention demanding? Eur J Neurosci. (2005) 22:1248–56. 10.1111/j.1460-9568.2005.04298.x 16176368

[19] Helmich RC de Lange FP Bloem BR Toni I. Cerebral compensation during motor imagery in Parkinson's disease. Neuropsychologia. (2007) 45:2201–15. 10.1016/j.neuropsychologia.2007.02.024 17448507

[20] Lewis GN Byblow WD Walt SE. Stride lenght regulation in Parkinson's disease: the use of extrinsic, visual clues. Brain. (2000) 123:2077–90. 10.1093/brain/123.10.2077 11004125

[21] Egerton T Williams DR Iansek R. Comparison of gait in progressive supranuclear palsy, Parkinson's disease and healthy older adults. BMC Neurol. (2012) 12:1–6. 10.1186/1471-2377-12-116 23031506 PMC3517411

[22] De Vos M Prince J Buchanan T FitzGerald JJ Antoniades CA. Discriminating progressive supranuclear palsy from Parkinson's disease using wearable technology and machine learning. Gait Posture. (2020) 77:257–63. 10.1016/j.gaitpost.2020.02.007 32078894

[23] Hatanaka N Sato K Hishikawa N Takemoto M Ohta Y Yamashita T. Comparative gait analysis in progressive supranuclear palsy and Parkinson's disease. Eur Neurol. (2016) 75:282–9. 10.1159/000445111 27288001

